# Supplementary material for: Neoadjuvant Treatment Lowers the Risk of Mesopancreatic Fat Infiltration and Local Recurrence in Patients with Pancreatic Cancer
Source: Cancers (Basel). 2021 Dec 23;14(1):68. doi: 10.3390/cancers14010068 (PMC8750596; doi:10.3390/cancers14010068)
Supplement: Supplementary file 1 [file cancers-14-00068-s001.zip › cancers-1498327-supplementary.pdf]

cancers

MDPI

Article

Neoadjuvant Treatment Lowers the Risk of Mesopancreatic Fat Infiltration and Local Recurrence in Patients with Pancreatic Cancer

Sami-Alexander Safi, Lena Haeberle, Alexander Rehders, Stephen Fung, Sascha Vaghiri, Christoph Roderburg, Tom Luedde, Farid Ziayee, Irene Esposito, Georg Fluegen and Wolfram Trudo Knoefel

Table S1. Dosage and intervalls of neoadjuvant regimes.

Neoadjuvant regime	Dosage	Intervall
Radiotherapy with	50 Gy and	1.8 Gy fractionized
Gemcitabine	300 mg/m2	1×/ week
Gemcitabine	1000 mg/m2	i.v. infusion 1×/ week
Nab-Paclitaxel	125 mg/m2	i.v. infusion 1×/ week
FOLFIRINOX	Oxaliplatin 85 mg/m2<br>Leukovorin 400 mg/m2<br>Irinotecan 180 mg/m2<br>Fluorouracil 400 mg/m2	i.v. infusion 1× every 2nd week

Gy: grey; mg: milligram.

Table S2: Correlation Analysis of patients stratified according to positive and negative mesopancreatic infiltration, n=27. There was a heterogenous distribution of CAP-treatment response across mesopancratic fat infriltation status. Correlation analysis was performed with the Pearson's test. ** indicates a p-value ≤ 0.01.

	No mesopancreatic fat infiltration n=10		Mesopancreatic fat infiltration n=17		p-value	r-value
Treatment response	n	%	n	%		
CAP 0	2	20.0	0	0	0.005**	+0.585**
CAP 1	4	40.0	1	5.9		
CAP 2	4	40.0	11	64.7		
CAP 3	0	0.0	5	29.4		

CAP: College of American Pathologists; CRM: circumferential resection margin
